# Trends and Disparities in Fall‐Related Head Injury Mortality Among Middle‐Aged and Older Adults (>55 Years) in the United States: A 21‐Year National Analysis (1999–2020)

**DOI:** 10.1002/brb3.71048

**Published:** 2025-11-10

**Authors:** Asad Ali Ahmed Cheema, F. N. U. Kritika, Amna Khan, Asad Khan, Aiman Sattar Memon, Syeda Maham Guftar Shah, Shayan Khan, Muhammad Mustafa, Abdul Raheem Malik, Fahad Ahmad Khan

**Affiliations:** ^1^ International School of Medicine International University of Kyrgyzstan Bishkek Kyrgyzstan; ^2^ General Surgery Indira Gandhi Institute of Medical Sciences Patna India; ^3^ Al‐Nafees Medical College, Isra University Islamabad Pakistan; ^4^ Bacha Khan Medical College Mardan Pakistan; ^5^ People's University of Medical and Health Sciences Nawabshah Pakistan; ^6^ Wah Medical College Wah Cantt. Pakistan; ^7^ Surgery Civil Hospital Karachi Pakistan; ^8^ Ziauddin Medical University Karachi Pakistan; ^9^ Continental Medical College Lahore Pakistan; ^10^ CMH Multan Institute of Medical Sciences Multan Pakistan

**Keywords:** demographics, fall, head injury, mortality, United States

## Abstract

**Background:**

Head injury is a leading cause of mortality from falls in adults aged ≥55 years. This study assessed demographic and geographic disparities in fall‐related head injury mortality among United States adults from 1999 to 2020.

**Methods:**

Mortality data were extracted from the Centers for Disease Control and Prevention Wide‐ranging Online Data for Epidemiologic Research (CDC WONDER) database using International Classification of Diseases (ICD‐10) codes for falls (W00–W19) and head injuries (S00–S09). Age‐adjusted mortality rates (AAMRs) per 100,000 population were calculated by year, sex, race/ethnicity, state, and urban–rural status. Trends were analyzed using Joinpoint regression to estimate annual percent change (APC). We refer to our cohort as “middle‐aged and older adults (≥55 years)” on the basis of epidemiological and trauma literature identifying midlife as the stage when fall risk and adverse head injury outcomes begin to rise. Furthermore, the World Society of Emergency Surgery (WSES) 2023 trauma guidelines explicitly recognize patients aged ≥55 years as a high‐risk population requiring special consideration, reinforcing the clinical relevance of this cutoff.

**Results:**

Between 1999 and 2020, 252,750 fall‐related head injury deaths occurred among adults aged ≥55 years. Most deaths occurred in medical settings (69.6%), followed by homes (9.2%), long‐term care (8.8%), and hospices (8.7%). AAMR nearly doubled, from 9.71 to 19.85, with a steeper rise from 1999 to 2007 (APC: 6.53) and slower growth thereafter (APC: 1.92). AAMRs were higher in men (22.1) than women (11.8), and highest in adults ≥85 years (81.7). Non‐Hispanic (NH) Whites had the highest AAMR (16.8), followed by NH Asians/Pacific Islanders (15.8), Hispanics (12.6), and NH Blacks (8.6). In 2020, state‐level AAMRs ranged from 9.5 (Alabama) to 24.0 (Wisconsin), with rural areas slightly exceeding urban ones (20.3 vs. 19.8).

**Conclusion:**

Fall‐related head injury mortality in US adults aged ≥55 years has steadily increased. Tailored prevention strategies are critical to reducing these preventable deaths, particularly in high‐risk groups.

## Graphical abstract

From 1999 to 2020, fall‐related head‐injury mortality among US adults ≥55 rose markedly, with the highest rates in men, the oldest ages, Midwestern states, and nonmetropolitan areas, underscoring widening disparities and the urgent need for targeted fall prevention and timely, equitable access to trauma care.

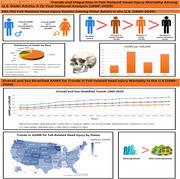

## 1 Introduction

Unintentional injuries, including falls, remain a major public health concern among middle‐aged and older adults (≥55 years) (Curry et al. 2011). In the United States, approximately 36 million falls occur annually, of which more than 14 million results in injuries, affecting nearly one in four older adults each year (CDC, Older Adult Fall Prevention 2024; MSD Manual Professional Edition 2025). These falls contribute to substantial morbidity and mortality, with around 27,000 deaths reported annually and numerous cases of traumatic injuries, including head trauma (MSD Manual Professional Edition 2025; Bergen et al. 2016). Falls are the leading cause of fatal injuries among middle‐aged and older adults and their consequences extend beyond fractures to include traumatic brain injuries (TBIs), which are associated with long‐term disability and death (CDC 2023). In 2021, there were approximately 69,473 TBI‐related deaths in the United States; the age‐adjusted TBI‐related mortality rate was approximately 19.5 per 100,000 population (Centers for Disease Control and Prevention 2023).

Older adults are particularly vulnerable due to age‐related physiological changes, including muscle weakness, impaired balance, and visual deficits (Shankar et al. 2017). Falls from beds or wheelchairs are more common among recurrent fallers, whereas walking‐related falls are most frequent overall and often result in bruises and head injuries (Hodgson et al. 2023; Berry and Miller 2008). Individuals with multiple comorbidities are at higher risk of mortality following fall‐related injuries, including those involving TBI (Xiong et al. 2019). Most falls occur at home, with smaller proportions occurring in outdoor or hospital settings (Kelsey et al. 2010). Temporal patterns have also been observed, with falls more frequent in winter, spring and on certain weekdays (López‐Soto et al. 2016). Risk factors also differ by sex: Men are more likely to fall due to pain and comorbidities, whereas depression and incontinence are stronger predictors in women (Ambrose et al. 2013).

Importantly, falls are largely preventable through evidence‐based interventions, risk assessment, environmental modifications, and public health initiatives. Programs such as Safe at Home (SAH) and the CDC's Stopping Elderly Accidents, Deaths, and Injuries (STEADI) initiative have been implemented to reduce fall risk among older adults (Markle‐Reid and Browne 2003; Centers for Disease Control and Prevention 2024; Ganz and Latham 2020). Despite these efforts, fall‐related mortality continues to rise, highlighting the need for updated epidemiological evidence (Peterson et al. 2018; Moreland et al. 2020).

Our findings extend and complement prior national surveillance. Peterson et al.^2022^ analyzed data and reported fall‐related TBI mortality trends in the United States from 2008 to 2017, showing the steepest increases among adults aged ≥75 years and in men (Peterson et al. 2022). Their analysis, although valuable for public health surveillance, was descriptive, limited to a decade, and used the CDC's broad TBI matrix case definition. Our work provides a more targeted and methodologically robust analysis of head injury–related mortality among middle‐aged and older adults, updating and extending the evidence base beyond previous CDC reports. We defined cases as deaths with head injury codes (International Classification of Diseases [ICD‐10] S00–S09) recorded in conjunction with an underlying cause of death classified as falls (W00–W19). This case definition isolates fall‐related head injury mortality, providing a clinically focused outcome distinct from the broader CDC TBI matrix.

This study examines nationwide two‐decade trends of mortality from falls‐related head injury among adults over 55 from 1999 to 2020 using Joinpoint regression to detect statistically significant changes in trend slopes. This approach captures long‐term shifts, including inflection points beyond 2017, and aligns the case definition more closely with clinical concerns in surgical and emergency medicine. By analyzing changes over time and evaluating variations across age groups, gender, and other demographic factors, this research aims to identify key risk patterns. The ultimate goal is to provide insights, strengthen prevention strategies, and improve healthcare interventions for older adults at risk of fall‐related head injuries.

## 2 Methods

### 2.1 Study Setting and Population

This study analyzed mortality trends from fall‐related head injuries among individuals aged ≥55 years using Centers for Disease Control and Prevention Wide‐ranging Online Data for Epidemiologic Research (CDC WONDER) mortality data. We selected 55 years as the lower cutoff because epidemiological and trauma research identifies this age as the point when physiologic vulnerability to falls, frailty, and adverse outcomes after head trauma begin to increase substantially. World Society of Emergency Surgery (WSES) 2023 Trauma guidelines also recognize ≥55 years as a threshold for high‐risk patients (De Simone et al. 2024). Accordingly, we refer to our study population as “middle‐aged and older adults (≥55 years),” which captures individuals at risk starting in midlife while ensuring comparability with studies restricted to adults aged ≥65 years. This publicly available and authoritative source includes death certificate records from all 50 states and the District of Columbia, spanning the years 1999–2020. The study utilized the 10th revision of ICD‐10 codes for falls (W00–W19) as the underlying cause of death. The same ICD‐10 codes have been previously validated and used to identify fall‐related head injuries in national mortality databases and injury surveillance systems (World Health Organization 2016; Stevens and Rudd 2015). Fall‐related deaths were classified as head injury‐related if the ICD‐10 multiple cause of death codes (S00–S09) indicated a diagnosis involving head trauma.

### 2.2 Data Abstraction

The study evaluated data on population size, year of death, location of death, demographic characteristics, urban–rural status, geographic region, and state. Demographic variables included sex, age, and race/ethnicity. The death location was categorized as a medical facility (including outpatient settings, emergency departments, inpatient settings, dead on arrival, or unspecified), hospice, or a nursing home/long‐term care facility. Race and ethnicity were grouped into the following categories: Non‐Hispanic (NH) White, NH Black or African American, Hispanic or Latino, NH Asian or Pacific Islander, and NH American Indian or Alaska Native. These classifications were based on self‐reported information from death certificates and aligned with prior studies using the CDC WONDER database. Urban–Rural classification scheme: Urban areas were categorized into two types: large metropolitan areas (populations of 1 million or more) and medium or small metropolitan areas (populations between 50,000 and 999,999). Rural areas were defined as counties with fewer than 50,000 residents, following the 2013 US Census classification. The geographic regions, including the Midwest, Northeast, South, and West, were designated according to US Census Bureau definitions (CDC/National Center for Health Statistics 2024; Ingram and Franco 2014).

### 2.3 Statistical Analysis

The crude and age‐adjusted mortality rates (AAMRs) per 100,000 population were used to assess deaths from fall‐related head injuries in the United States between 1999 and 2020. These rates were stratified by year, sex, race/ethnicity, urban–rural classification, and state and were reported with 95% confidence intervals (CIs). AAMRs were standardized to the 2000 US population to allow for comparability over time (Anderson and Rosenberg 1998). To evaluate trends in AAMRs, Joinpoint regression analysis (Joinpoint Regression Program, version 4.9.0.0, National Cancer Institute) was conducted to estimate the annual percent change (APC) with 95% CI (National Cancer Institute 2025). Joinpoint regression identifies significant changes in mortality trends by fitting log‐linear regression models and detecting points where the rate of change shifts. This approach is particularly suitable given the expected changes in population parameters over the study period, including demographic shifts, increasing prevalence of comorbidities, and evolving living environments among adults aged ≥55 years, which may influence fall‐related head injury risk. APCs were considered statistically significant if the corresponding slope differed from zero (*p* < 0.05) based on two‐tailed *t*‐tests.

### 2.4 Protocol Approval and Patient Consent

As the dataset is de‐identified and publicly accessible from CDC WONDER database, the study did not require approval from an Institutional Review Board (IRB) or ethics committee. The reporting of this observational study adhered to the STROBE (Strengthening the Reporting of Observational Studies in Epidemiology) guidelines and the STROBE reporting checklist when editing, included in Supporting Information A (von Elm et al. 2007, 2025).

## 3 Results

### 3.1 Annual Trends of AAMR

**FIGURE 1:**
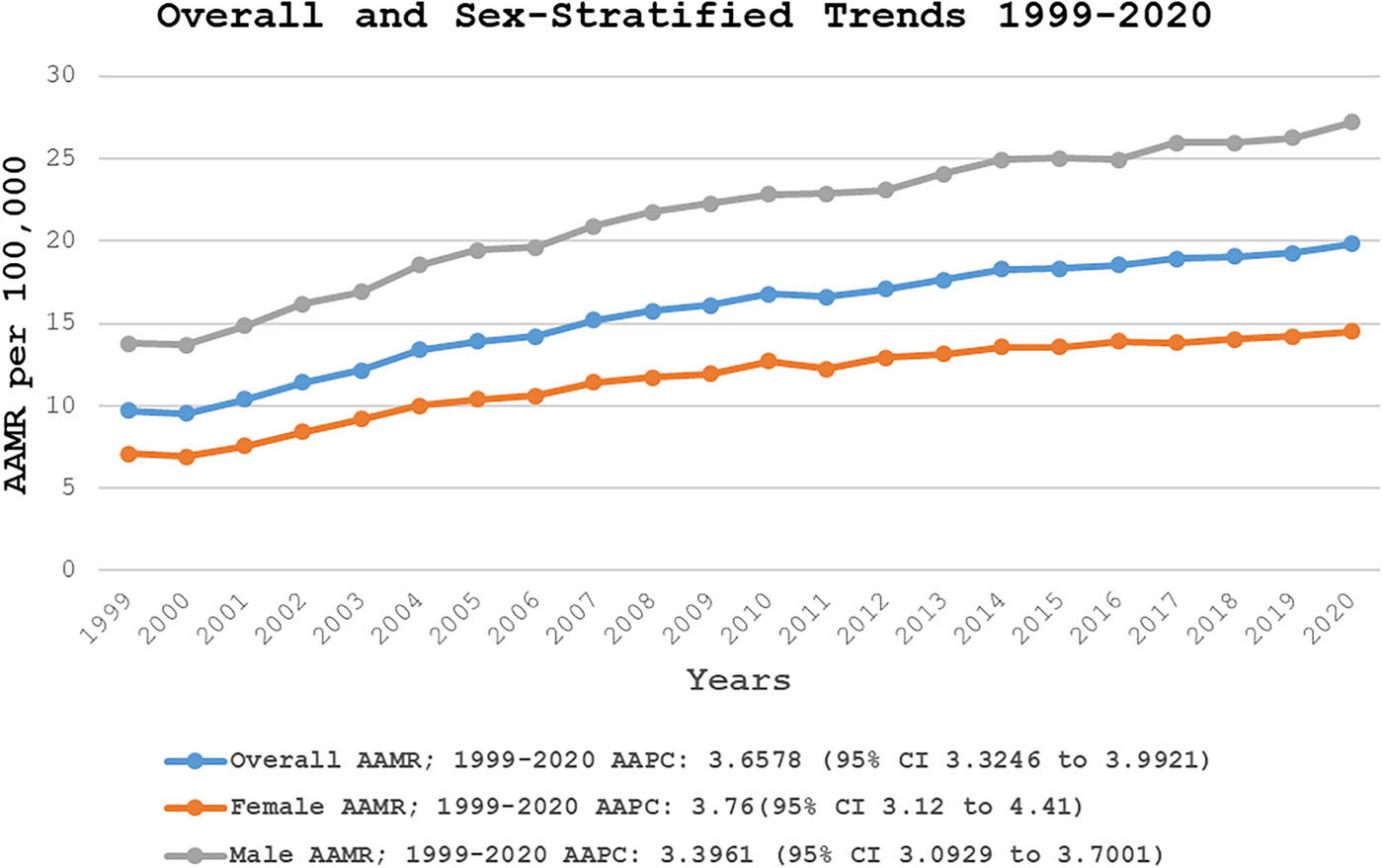
Overall and sex‐stratified fall‐related head injury age‐adjusted mortality rates per 100,000 adults in the United States, 1999–2020. AAMR, age‐adjusted mortality rate; CI, confidence interval.

The overall AAMR for fall‐related head injury deaths was 9.71 per 100,000 in 1999 and increased to 19.85 per 100,000 in 2020 (Central Illustration, Table S2). The overall AAMR increased significantly from 1999 to 2007 (APC: 6.53; 95% CI: 5.68–7.38), followed by a continued but tapered rise from 2007 to 2020 (APC: 1.92; 95% CI: 1.65–2.20) (Figure 1, Tables S1 and S10).

### 3.2 Stratification by Sex

Older men consistently had higher AAMRs than older women throughout the study period (overall AAMR for men: 22.1; 95% CI: 21.98–22.22; for women: 11.77; 95% CI: 11.7–11.84). In 1999, the AAMR for older men was 13.80 (95% CI: 13.30–14.30), increasing to 20.90 in 2007 (APC: 5.95; 95% CI: 5.19–6.73). This followed a continued but subtle upward trend to 27.22 until 2020 (APC: 1.85; 95% CI: 1.60–2.10). Similarly, the AAMR for older women in 1999 was 7.10 (95% CI: 6.83–7.38), rising to 9.99 in 2004 (APC: 8.25; 95% CI: 6.15–10.38). It continued to increase, reaching 12.70 in 2010 (APC: 4.02; 95% CI: 2.42–5.64), followed by a further but more gradual rise to 14.50 (95% CI: 14.17–14.83) in 2020 (APC: 1.44; 95% CI: 0.98–1.91) (Figure 1, Tables S1, S2, and S10).

### 3.3 Stratification by Race/Ethnicity

**FIGURE 2:**
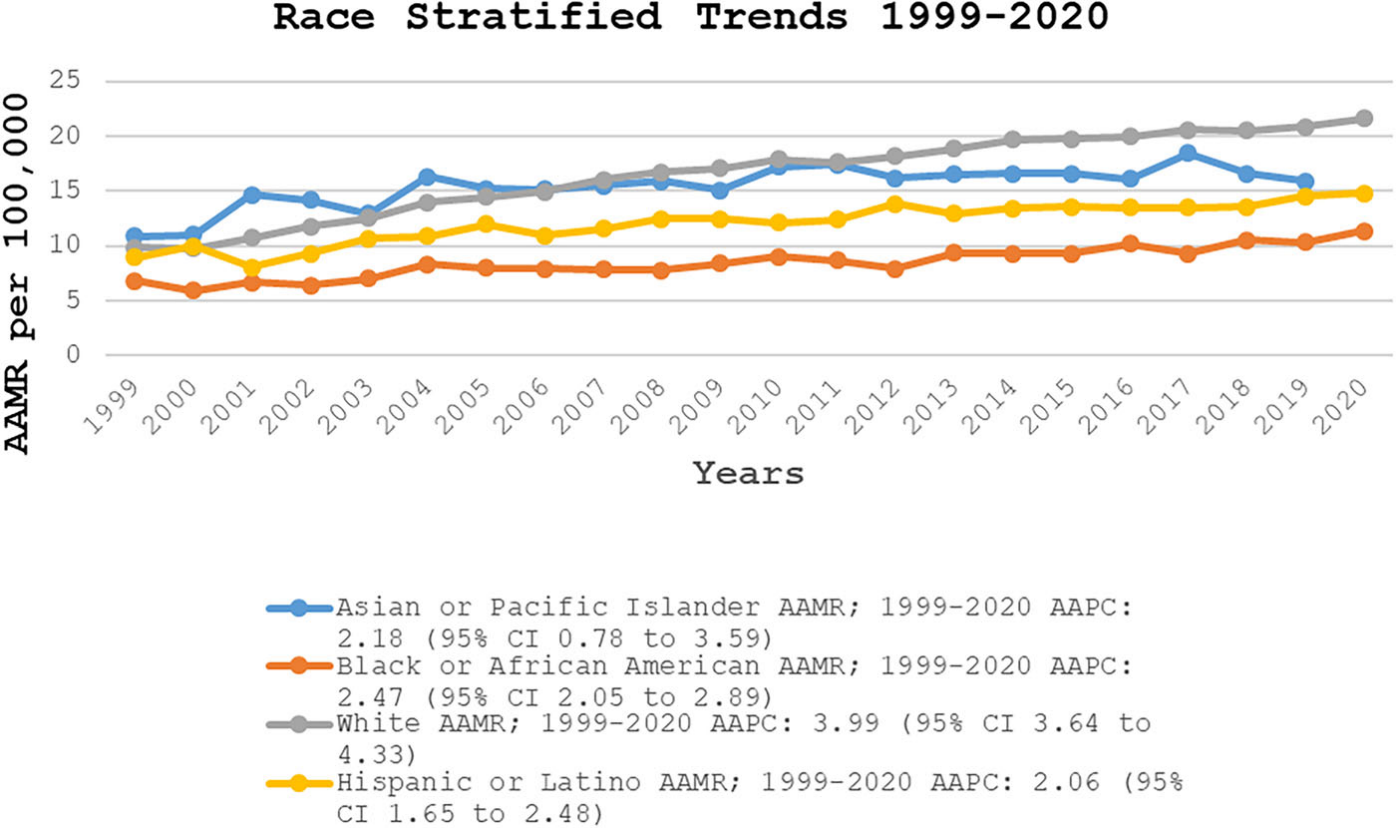
Race/ethnicity‐stratified fall‐related head injury age‐adjusted mortality rates per 100,000 adults in the United States, 1999–2020. AAMR, age‐adjusted mortality rate; CI, confidence interval.

When stratified by race/ethnicity, AAMRs were highest among NH White patients, followed by NH Asian or Pacific Islanders, Hispanic or Latino, and NH Black or African American populations (overall AAMR NH White: 16.87; 95% CI: 16.8–16.94; NH Asian or Pacific Islanders: 15.86; 95% CI: 15.52–16.19; Hispanic or Latino: 12.65; 95% CI: 12.44–12.87; NH Black or African American: 8.67; 95% CI: 8.51–8.83). Among NH White individuals, the AAMR increased sharply to 16.05 in 2007 (APC: 7.01; 95% CI: 6.15–7.89), followed by a continued but slower increase until 2020 (APC: 2.17; 95% CI: 1.88–2.46). For Hispanic or Latino and NH Black or African American populations, AAMRs exhibited a steady upward trend from 1999 to 2020 (Hispanic or Latino APC: 2.06; 95% CI: 1.65–2.48; NH Black or African American APC: 2.47; 95% CI: 2.05–2.89). In contrast, NH Asian or Pacific Islanders experienced an initial increase in AAMR from 1999 to 2005 (APC: 6.29; 95% CI: 1.26–11.57), followed by a period of decline until 2020 (APC: 0.58; 95% CI: −0.15 to 1.31), reaching an AAMR of 15.09 in 2020 (95% CI: 13.47–16.71) (Figure 2, Tables S1, S3, and S10).

### 3.4 Stratification by Age

**FIGURE 3:**
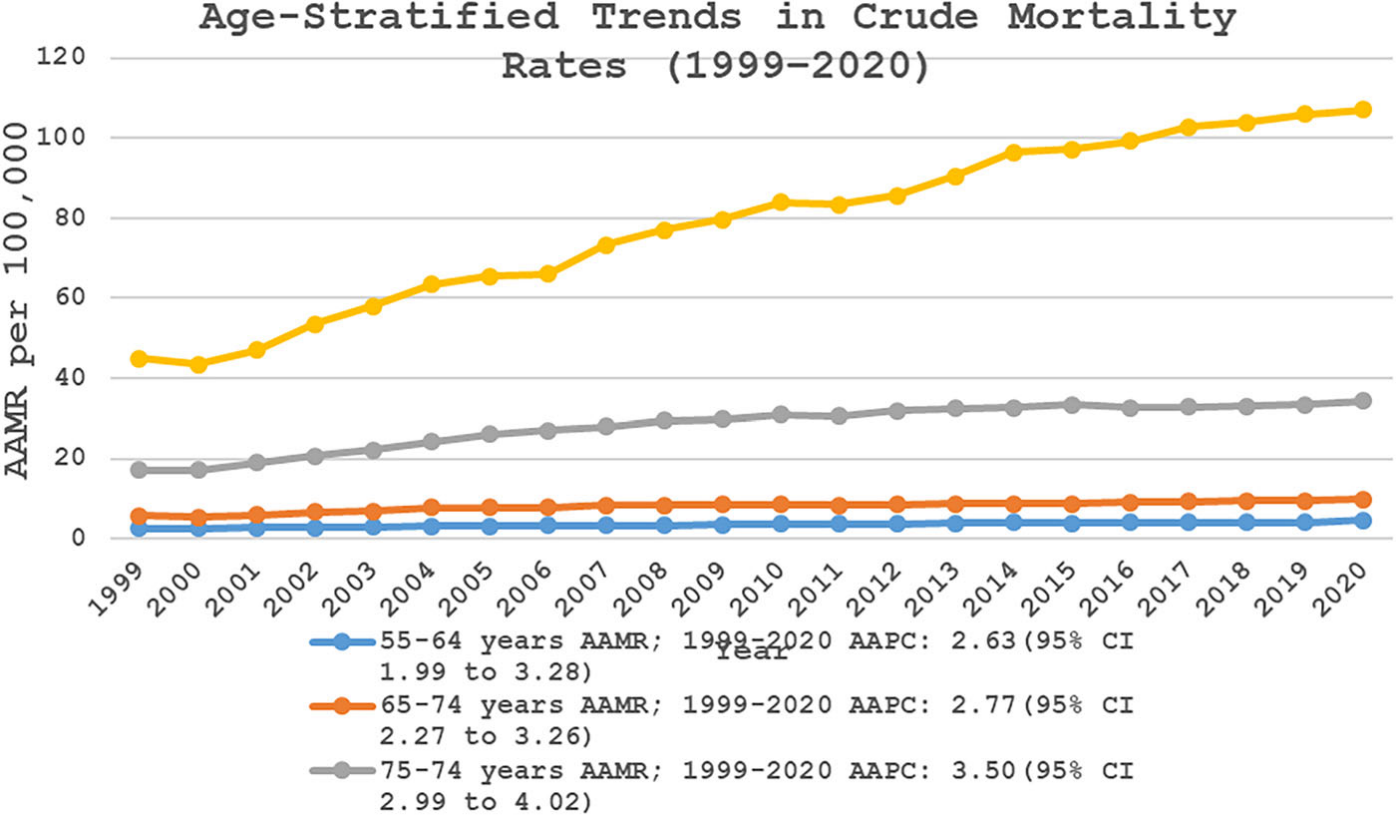
Age‐stratified fall‐related head injury crude mortality rates per 100,000 adults in the United States, 1999–2020. AAMR, age‐adjusted mortality rate; CI, confidence interval.

Overall Crude mortality rates were highest in the older age groups, with the highest rate observed in the 85+ years age group (CMR: 81.73; 95% CI: 81.22–82.24), followed by the 75–84 years age group (CMR: 28.44; 95% CI: 28.25–28.63), the 65–74 years age group (CMR: 8.32; 95% CI: 8.24–8.40), and the 55–64 years age group (CMR: 3.61; 95% CI: 3.57–3.66). The temporal trend analysis reported a consistent increase in CMR due to fall‐related head injuries across all age groups. The 85+ year age group exhibited the highest mortality rates, with a significant rise from 1999 to 2008 (APC: 6.80, 95% CI: 5.75–7.85), followed by a slower but continued increase from 2008 to 2020 (APC: 2.85; 95% CI: 2.41–3.29) (CMR: 107.02; 95% CI: 104.54–109.51). Similarly, the 75–84 years age group experienced a sharp increase in mortality from 1999 to 2005 (APC: 7.90; 95% CI: 6.72–9.10), which then slowed from 2005 to 2010 (APC: 3.65; 95% CI: 1.89–5.44) and further decelerated from 2010 to 2020 (APC: 0.88; 95% CI: 0.50–1.26) (CMR: 34.24; 95% CI: 33.35–35.13). The 65–74 age group showed a significant increase from 1999 to 2005 (APC: 6.48; 95% CI: 4.73–8.26), followed by a more gradual rise from 2005 to 2020 (APC: 1.32; 95 % CI: 1.01–1.63) (CMR: 9.81; 95% CI: 9.47–10.15). Similarly, the 55–64 years age group experienced an initial rise from 1999 to 2007 (APC: 3.93; 95% CI: 2.35–5.53), followed by a continued but slower increase from 2007 to 2020 (APC: 1.84; 95% CI: 1.28–2.40) (CMR: 4.62; 95% CI: 4.41–4.82) (Figure 3, Tables S1, S4, and S10).

### 3.5 Stratification by Census Region

**FIGURE 4:**
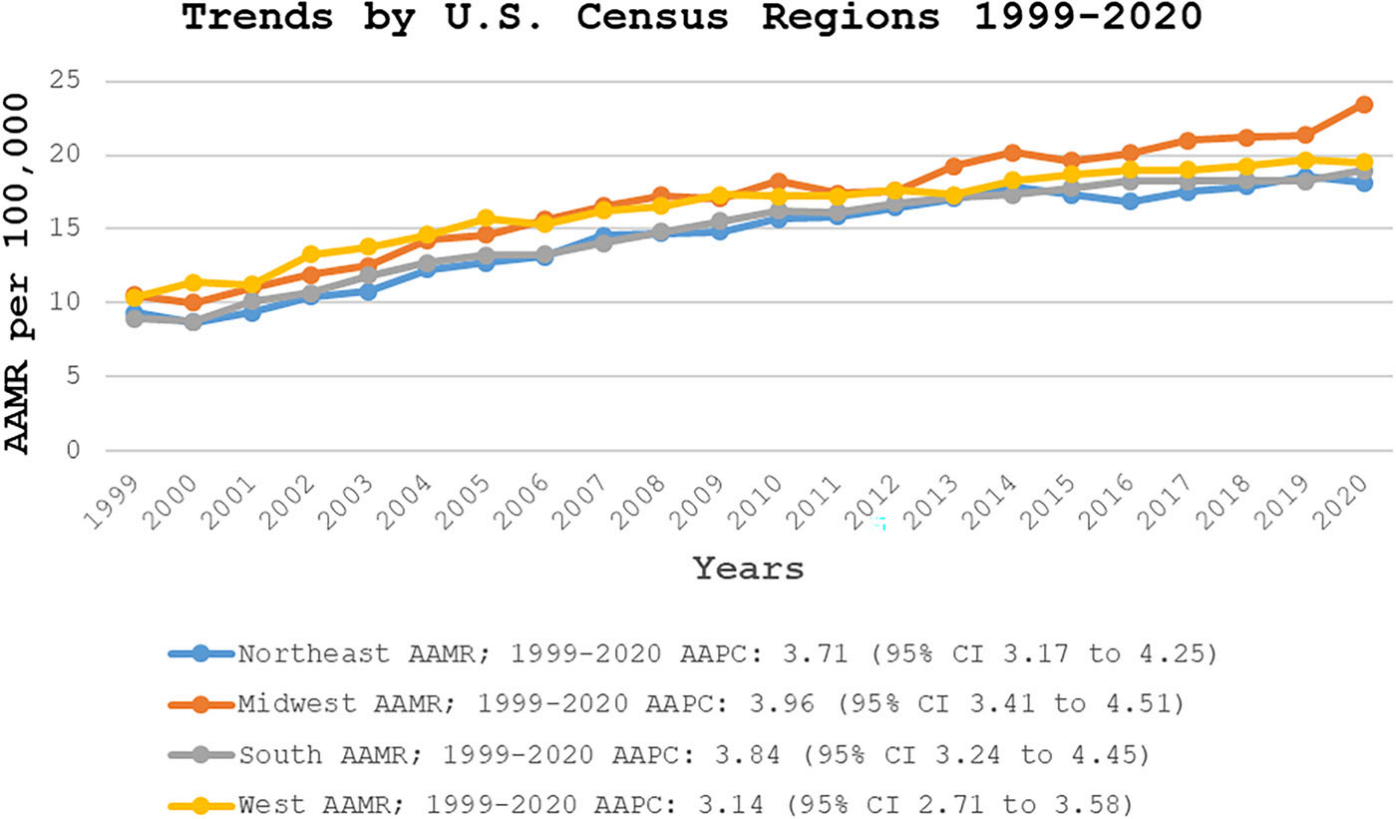
Region‐stratified fall‐related head injury age‐adjusted mortality rates per 100,000 adults in the United States, 1999–2020. AAMR, age‐adjusted mortality rate; CI, confidence interval.

Throughout the study period the highest mortality was observed in the Midwestern region (AAMR: 17.19; 95% CI: 17.06–17.33), followed by Western (AAMR: 16.77; 95% CI: 16.63–16.91), Southern (AAMR: 15.36; 95% CI: 15.26–15.46), and Northeastern (AAMR: 14.81; 95% CI: 14.68–14.94) regions (Figure 4, Table S5).

### 3.6 Stratification by Urbanization

**FIGURE 5:**
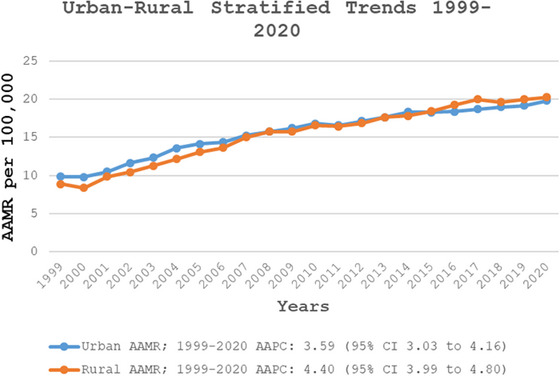
Urban and rural stratified fall‐related head injury age‐adjusted mortality rates per 100,000 adults in the United States, 1999–2020. AAMR, age‐adjusted mortality rate; CI, confidence interval.

Metropolitan areas had higher overall AAMRs than nonmetropolitan areas (AAMR: 16.01; 95% CI: 15.95–16.08 vs. AAMR: 15.62; 95% CI: 15.48–15.77). However, this trend reversed after 2011, with nonmetropolitan areas surpassing metropolitan areas in mortality rates by 2020 (AAMR: 20.28; 95% CI: 19.55–21.00 vs. AAMR: 19.82; 95% CI: 19.50–20.14). In the metropolitan area, AAMRs showed a significant increase from 1999 to 2004 (APC: 7.40; 95% CI: 5.72–9.10), followed by a slower but continued increase through 2009 (APC: 4.07; 95% CI: 2.20–5.98), with continued but more gradual growth until 2020 (APC: 1.69, 95% CI: 1.38–2.01). Similarly, in nonmetropolitan areas, AAMRs increased from 1999 to 2007 (APC: 7.40; 95% CI: 6.60–8.66), followed by a sustained upward trend through 2020, although at a slower rate (APC: 2.45; 95% CI: 2.11–2.80) (Figure 5, Tables S1, S6, and S10).

### 3.7 Stratification by Place of Death

A total of 252,750 fall‐related head injury deaths occurred among older adults (aged ≥55 years) between 1999 and 2020 (Table S7). Information on the location of death was available for 243,373 deaths. Of these, 69.6% occurred within medical facilities, 9.21% at home, 8.82% in nursing homes or long‐term care facilities, and 8.65% in hospices (Figure S1, Table S8).

### 3.8 Stratification by State

A significant difference in AAMR was observed in different states, with the AAMR ranging from 9.50 (95% CI: 9.12–9.89) in Alabama to 24.03 (95% CI: 23.49–24.58) in Wisconsin. States in the top 90th percentile, including Wisconsin, Vermont, Washington, Colorado, South Dakota, Minnesota, Hawaii, and Utah, had approximately 2.5 times the AAMRs compared to those in the bottom 10th percentile, namely, Alabama, New Jersey, and Louisiana (Figure S2, Table S9).

## 4 Discussion

Our analysis of 20 years of data from the CDC WONDER database revealed several key findings regarding fall‐related head injury mortality among adults aged 55 years and older in the United States between 1999 and 2020. Although ≥65 years is conventionally used to define older adults, we analyzed adults aged ≥55 years to capture both middle‐aged and older adults. This choice reflects evidence that vulnerability to falls and poor outcomes increases beginning in the mid‐50s, and is further supported by the WSES 2023 trauma guidelines, which identify ≥55 years as a high‐risk threshold for trauma patients. We acknowledge this methodological distinction while noting that our subgroup analyses (55–64, 65–74, 75–84, and ≥85) allow comparability with studies restricted to ≥65 years (De Simone et al. 2024). In our study, first, the mortality rates showed a marked increase from 1999 to 2007, followed by a steady and sustained rise from 2007 through 2020. Second, NH White adults had the highest fall‐related head injury AAMR compared with other racial groups in the same age range of 55 years and older. Third, there was a notable variation in data across different regions, with Wisconsin having the highest. The states that were placed in the top 90th percentile (Wisconsin, Vermont, Washington, Colorado, South Dakota, Minnesota, Hawaii, and Utah) had approximately double the AAMRs compared with states placed in the lower 10th percentile (Alabama, New Jersey, and Louisiana). Furthermore, nonmetropolitan areas had a higher mortality rate than metropolitan areas from 2011 to 2020 (AAMR 8.7 vs. 6.6).

The overall AAMR for fall‐related head injuries more than doubled over the study period, increasing from 9.71 in 1999 to 19.85 in 2020. The sharpest rise occurred between 1999 and 2007, followed by a slower but continuous increase from 2007 to 2020. This initial surge in mortality likely reflects a combination of factors, including rapid demographic shifts with a growing population of middle‐aged and older adults, improvements in death certificate reporting, increased completeness of multiple cause‐of‐death data, and changes in ICD coding practices, all of which may have led to more accurate capture of fall‐related head injury deaths during this period (CDC, Older Adult Fall Prevention 2024; CDC 2023,; Peterson et al. 2018; Santos‐Lozada 2023). The continued, albeit slower, increase in fall‐related head injury mortality post‐2007 may reflect the partial effectiveness of national public health interventions aimed at fall prevention. Key initiatives launched during this period include the CDC's STEADI initiative, introduced in 2012; CMS quality measures targeting fall prevention in healthcare settings; and the Healthy People 2020 goals, which prioritized injury prevention in middle‐aged and older adults (Centers for Disease Control and Prevention 2024; Centers for Medicare and Medicaid Services 2024; US Department of Health and Human Services 2024). Additionally, community‐based programs such as the Otago Exercise Program and A Matter of Balance, supported by the National Council on Aging, likely contributed to improved balance, strength, and awareness of falls among adults aged 55 or above. Together, these programs may have mitigated a sharper rise in mortality, despite the aging population and increasing incidence of falls. Nevertheless, the persistent increase in fall‐related head injury mortality indicates that current prevention efforts, although beneficial, remain insufficient. There is a critical need for more robust and evidence‐based strategies to mitigate this growing public health concern effectively.

Our findings align with those of Santos‐Lozada, who reported increasing fall‐related mortality among US adults aged ≥65 years from 1999 to 2020 using the same CDC WONDER data (CDC 2023; Santos‐Lozada 2023). Although both studies demonstrate consistent upward trends, our analysis extends this work by focusing specifically on fall‐related head injury mortality and including adults aged ≥55 years, thereby capturing a younger at‐risk population and a clinically distinct outcome.

Crude and AAMRs were calculated per 100,000 population, with denominators obtained from US Census Bureau estimates stratified annually by age group, sex, race/ethnicity, and urban–rural classification (CDC 2023). Adults aged ≥85 years had the highest AAMRs, exceeding those of the 55–64 and 65–74 age groups. This aligns with findings from other studies reporting that increasing age is strongly associated with worse outcomes following traumatic brain injury (TBI). Specifically, adults aged 55 and older who present with severe TBIs have reported mortality rates ranging from 30% to 80%, depending on injury severity, physiological reserve, and comorbidities. Compared to younger patients, middle‐aged and older adults often experience delayed presentation, diminished cerebral autoregulation, and impaired physiologic responses to injury, all of which contribute to poor outcomes (Hukkelhoven et al. 2003; Mosenthal et al. 2002; Pennings et al. 1993; Timiras 2002). Falls account for the majority (51%) of trauma‐related injuries among adults aged 55 or above, making them the leading cause, whereas motor vehicle traffic accidents rank second, contributing to 9% of such injuries (Thompson et al. 2006). A study showed that motor vehicle collision‐related injuries totaled 154,000 in middle‐aged and older adults in the United States in 2002 (United States. Department of Transportation. National Highway Traffic Safety Administration 2004). Head computed tomography (CT) in some older adults may reveal moderate cerebral atrophy, which can mask underlying abnormalities even when the initial neurological examination appears normal (Mack et al. 2003). A study investigating cerebral autoregulation in older adults following TBI utilized Transcranial Doppler imaging along with measurements of cerebral perfusion pressure (CPP) and intracranial pressure (ICP) to evaluate autoregulation and pressure reactivity indices. Findings showed that ICP significantly declined with age, resulting in a corresponding increase in CPP, indicating diminished cerebrovascular responsiveness in the aging brain (Svedung Wettervik et al. 2025). More comorbidities, such as dementia or mild cognitive impairment, directly contribute to a higher risk of head injuries within the aging population in the United States (Plassman et al. 2000).

A significant disparity was observed between men and women, with older men consistently experiencing greater mortality rates than older women, with the overall AAMR for men nearly double that of women. Some studies suggest that estrogen and progesterone may improve TBI outcomes in women by supporting cerebral perfusion, but conflicting research makes this uncertain (Davis et al. 2006). These hormones have also been shown to decrease pro‐inflammatory cytokines, such as IL‐6, IL‐1β, TGF‐β, and TNF‐α, which are known to drive inflammation. Elevated levels of these cytokines in cerebrospinal fluid and brain tissue can worsen trauma‐induced brain lesions (Ross et al. 1994). The secondary damage resulting from hypoperfusion of the brain, lipid peroxidation, and free radical production triggered by ischemia is also reduced by progesterone's neuroprotective and anti‐lipid peroxidation function (Roof et al. 1997). The incidence of traumatic injuries in men tends to be high in the youth, middle‐aged, and prime adulthood. At the same time, the proportion of women increases significantly with age in the elderly and the very old (Faul et al. 2010). The potential neuroprotective role of these hormones remains unclear (Eom et al. 2021). According to a study, men had higher rates of skull fractures, AEDH, DAI, TICH, and CSDH, whereas women were more frequently affected by concussion, ASDH, and TSAH (Eom et al. 2021). A comprehensive CDC report highlights that men have higher age‐adjusted rates of TBI‐related emergency department visits, hospitalizations, and deaths than women across all age groups. The data underscore the disproportionate impact of TBIs on older men, particularly due to falls (Faul et al. 2010). Another study examined the epidemiology and outcomes of TBI in older adults, noting that men are at a higher risk for sustaining TBIs and experiencing worse outcomes compared to women. Factors contributing to this disparity include a higher likelihood of engaging in riskier activities and differences in comorbid conditions (Ambrose et al. 2013). An article examining the epidemiology of falls in older adults highlights various risk factors, including male gender, that are associated with a greater likelihood of serious injuries such as TBIs. The study underscores that older men are at a higher risk of experiencing severe outcomes from falls, which contributes to elevated mortality rates in this group (Ambrose et al. 2013). Another consideration is the role of competing risks. Women, who live longer on average, may be more likely to die of other fall‐related injuries such as hip fractures or of comorbid conditions, thereby reducing the proportion of deaths attributed to head injuries. This limitation highlights that sex differences in fall‐related head injury mortality may partly reflect differential competing risks rather than intrinsic differences in head trauma outcomes (Stevens and Rudd 2015; Fine and Gray 1999).

The analysis revealed significant racial and ethnic disparities in fall‐related head injury mortality. NH White individuals had the highest AAMR. Significant racial and ethnic disparities exist in fall‐related head injury mortality in the United States. NH White adults, particularly those aged ≥75, experience the highest AAMRs from fall‐related head injuries, primarily due to demographic factors such as advanced age, higher prevalence of osteoporosis and comorbidities, and a greater likelihood of living alone, which increases post‐fall complications and delays in care (Rubenstein 2006; Berry et al. 2010). In 2020, NH White adults aged ≥85 had an unintentional fall death rate of 334.8 per 100,000, the highest among all racial and ethnic groups (Daugherty et al. 2019). Although American Indian/Alaska Native populations show the highest overall TBI‐related death rates, these are more often linked to diverse causes such as assaults and vehicle incidents. In contrast, unintentional falls are the predominant cause of TBI‐related deaths in older NH White populations. Meanwhile, racial and ethnic minorities, including Black and Hispanic groups, tend to report poor long‐term functional outcomes and community reintegration post‐TBI despite lower mortality from falls (Berry et al. 2010).

The mortality rates varied significantly across states, with Wisconsin reporting the maximum AAMR (24.03) and Alabama the minimum (9.50). Wisconsin ranks highest in US fall‐related deaths among middle‐aged and older adults, influenced by icy winters, alcohol use, and strong reporting systems (Centers for Disease Control and Prevention [CDC] 2023). The highest mortality burden was noticed in the Midwest, followed by the West, South, and Northeast. The Midwest has the highest fall‐related head injury AAMRs, likely due to its older population, icy weather that increases fall risk, and limited access to trauma care in rural areas, compared to warmer, more urbanized states with younger populations (Garnett et al. 2022; Peterson et al. 2022; Kakara et al. 2023).

Additionally, the study found that metropolitan areas initially had higher mortality rates than nonmetropolitan areas. Still, after 2011, this trend reversed, with nonmetropolitan areas surpassing metropolitan areas in mortality rates (AAMR 8.7 vs. 6.6). A study suggests that all‐cause mortality from TBI is 23% higher in rural areas compared to urban areas in the United States (Kuehn 2020; Brown et al. 2019). The implementation of the Brain Trauma Foundation's widely recognized guidelines for TBI management has helped reduce TBI‐related mortality (Kakara et al. 2023). Moreover, the increasing burden in rural areas may be due to limited healthcare access, delayed emergency response times, and differences in fall prevention measures (Kuehn 2020; Brown et al. 2019). Many rural areas lack fall prevention programs and timely access to emergency imaging, such as CT scans. This is critical, as rapid diagnosis of conditions like SAH or ICH is essential for effective intervention. Delays in neuroimaging and transfers from rural hospitals further increase patient risk (Stevens et al. 2014; Watchorn et al. 2021).

The rise in fall‐related head injury mortality among adults aged ≥55 years may result from increased fall incidence, higher likelihood of head injury per fall, or worsening outcomes after injury. Although our analysis focuses on fatal head injuries, evidence suggests that multifactorial risk factors, including age‐related physiological changes, comorbidities, and environmental hazards, contribute to both falls and injury severity in older adults (Fleming et al. 2014). In addition, increasing survival with chronic conditions such as cardiovascular disease, diabetes, and dementia means that more adults are living long enough to experience frailty, recurrent falls, and their complications, which likely contributes to the time‐related rise in fall‐related head injury mortality (Hu and Baker 2010). Prevention strategies should therefore address both fall risk and injury mitigation to reduce mortality effectively.

Overall, the findings suggest that fall‐related head injuries will remain a growing concern as the population ages. Proactive measures are needed to deal with this rising trend and reduce preventable deaths.

### 4.1 Future Directions and Path Forward

Our findings underscore several immediately actionable directions. First, expand and target fall‐prevention programs (e.g., medication review and deprescribing, vision correction, strength/balance training, and home hazard mitigation) to men, adults ≥85 years, and nonmetropolitan counties, where mortality is highest. Second, integrate STEADI workflows into routine primary care and emergency/trauma encounters, with electronic health record (EHR) flags for recent falls, sedative polypharmacy, or cognitive impairment. Third, strengthen rural trauma capacity by shortening prehospital times (emergency medical services [EMS] triage protocols, tele‐trauma consults), ensuring rapid CT availability, and facilitating time‐to‐neurosurgery transfer. Fourth, test community‐level interventions (winter sidewalk maintenance, fall‐safe housing retrofits, community exercise programs) using policy or natural‐experiment designs. Finally, future research should link National Vital Statistics System (NVSS) mortality with National Emergency Medical Services Information System (NEMSIS), hospital claims/encounters from the Healthcare Cost and Utilization Project (HCUP), and area‐level socioeconomic datasets to (i) separate injury occurrence from post‐injury survival, (ii) measure TBI severity, and (iii) evaluate equity impacts. Analytically, competing‐risk models, age‐period‐cohort decomposition, and interrupted time‐series for pandemic and policy periods can clarify mechanisms and guide resource allocation.

## 5 Limitations

Although this study offers insightful perspectives on fall‐related head injury mortality trends among middle‐aged and older adults, several limitations should be acknowledged. First, the study is based on mortality data from death certificates, which may be subject to misclassification or inaccurate reporting of the cause of death. Second, the data do not include detailed information on contributing factors such as preexisting medical conditions (e.g., osteoporosis, dementia, and frailty), medication use, or specific circumstances leading to falls. These factors may play a significant role in explaining differences in mortality rates. This study focuses only on fatal fall‐related head injuries. Third, it does not account for nonfatal falls, which are far more common and significantly burden healthcare systems. The exclusion of nonfatal cases limits understanding of the full scope of fall‐related injuries. Using CDC WONDER mortality data, only the place of death is recorded. Therefore, we are unable to report the location of the fall itself (e.g., home and public space), which may be relevant for understanding fall circumstances and targeting prevention efforts. Differences in healthcare access, quality of care, and availability of fall prevention programs across states and between metropolitan and nonmetropolitan areas may contribute to variations in mortality rates. The study does not differentiate between deaths occurring immediately after a fall versus those occurring later due to complications (e.g., infections and prolonged immobility). Understanding the timing of death could provide deeper insights into potential intervention points. The increasing aging population over the study period may contribute to the rising mortality rates observed. However, this study does not account for changes in population structure, life expectancy, or improvements in medical care that may have influenced the results. Over the study period, advancements in trauma care, neurosurgery, and intensive care may have influenced mortality trends. However, this study does not assess the impact of these medical advancements on survival rates following fall‐related head injuries.

This analysis could not incorporate linked, person‐level datasets such as the NVSS, NEMSIS, or HCUP. Without these linkages, it is not possible to distinguish whether increasing mortality reflects higher fall incidence or poorer post‐injury survival. Furthermore, area‐level socioeconomic, environmental, and policy factors that shape fall risk and outcomes were unavailable. Joinpoint regression assumes log‐linear trend segments and may oversimplify nonlinear temporal dynamics, including the effects of ICD coding changes or pandemic‐era disruptions (2019–2020). Future studies should integrate NVSS‐NEMSIS‐HCUP data and employ age‐period‐cohort and competing‐risk models to better understand structural and clinical contributors to the observed disparities.

## 6 Conclusion

The results demonstrated a steady increase in the AAMR of fall‐related head injuries from 1999 to 2020. By the end of 2020, the highest AAMR was observed among NH White adults, men, residents of the Midwestern region, and nonmetropolitan areas. A rise in AAMR was noted across all racial/ethnic groups, except for NH Asians or Pacific Islanders, who experienced an increase until 2005, followed by a decline until 2020. The Northeastern region of the United States had the lowest AAMR by the end of the study period. Additionally, the study reveals an age‐related increase in fall‐related head injury mortality, with the highest crude death rates observed in adults aged 85 years and older. The increasing mortality trends across all demographic groups highlight the urgent need for enhanced fall prevention measures, improved access to healthcare, and targeted interventions to mitigate fall‐related risks, particularly among the most vulnerable populations. Healthcare providers must prioritize strategies to address this growing public health issue and enhance the safety and well‐being of aging populations.

## Author Contributions

**Asad Ali Ahmed Cheema**: conceptualization, data curation, formal analysis, supervision, writing – original draft, writing – review and editing. **F. N. U. Kritika**: investigation, writing – original draft, data curation. **Amna Khan**: methodology, formal analysis, writing – review and editing. **Asad Khan**: investigation. **Aiman Sattar Memon**: data curation. **Syeda Maham Guftar Shah**: visualization. **Shayan Khan**: resources. **Muhammad Mustafa**: validation. **Abdul Raheem Malik**: project administration. **Fahad Ahmad Khan**: software.

## Funding

The authors have nothing to report.

## Ethics Statement

This study used publicly available, de‐identified data and did not require formal ethics approval.

## Consent

The authors have nothing to report.

## Conflicts of Interest

The authors declare no conflicts of interest.

## Supporting information



Supplementary Materials: brb371048‐sup‐0001‐SuppMat.docx

## Data Availability

This study used publicly available data from the Centers for Disease Control and Prevention Wide‐ranging Online Data for Epidemiologic Research (CDC WONDER) database, accessible at https://wonder.cdc.gov. Additional details about the data processing and analysis are available from the corresponding author upon reasonable request.
