# Supplementary material for: Trends and Disparities in Fall‐Related Head Injury Mortality Among Middle‐Aged and Older Adults (>55 Years) in the United States: A 21‐Year National Analysis (1999–2020)
Source: Brain Behav. 2025 Nov 10;15(11):e71048. doi: 10.1002/brb3.71048 (PMC12602457; doi:10.1002/brb3.71048)

**Topic: Trends and Disparities in Fall-Related Head Injury Mortality Among Older Adults in the United States A 21-Year National Analysis (1999–2020)**

**QUERY CRITERIA ;**

**AGE GROUP: +55**

**AAMR PER 100,000**

**OVERALL AAPC: 3.6578*(P-VALUE < 0.000001)**

**TOTAL DEATHS : 252750**

**SIGNIFICANT INCREASE IN AAMR:**

**1999: 9.71**

**2020: 19.85**

**SUPPLEMENT A:** STROBE Statement

|  | Item No. | Recommendation | Page  No. | Relevant text from manuscript |
| --- | --- | --- | --- | --- |
| **Title and abstract** | 1 | (*a*) Indicate the study’s design with a commonly used term in the title or the abstract | 5-6 | “Mortality trends in Fall-related Head injury... A CDC WONDER Analysis” |
|  |  | (*b*) Provide in the abstract an informative and balanced summary of what was done and what was found | 5-6 | Abstract includes objectives, methods (data source, analysis), key results (AAMR trends, disparities), and conclusions. |
| Introduction | | | |  |
| Background/rationale | 2 | Explain the scientific background and rationale for the investigation being reported | 7 | Introduction discusses Fall/Head injury burden, Falls as a risk factor, and parallel mortality trends. |
| Objectives | 3 | State specific objectives, including any prespecified hypotheses | 7 | To assess demographic and regional disparities in hypertension and dementia-related mortality across the U.S. |
| Methods | | | |  |
| Study design | 4 | Present key elements of study design early in the paper | 8-9 | “The CDC WONDER database was used... STROBE guidelines were followed" |
| Setting | 5 | Describe the setting, locations, and relevant dates, including periods of recruitment, exposure, follow-up, and data collection | 8-9 | "US deaths from 1999-2020," "CDC WONDER database," "National Center for Health Statistics Urban-Rural Classification" |
| Participants | 6 | (*a*) *Cohort study*—Give the eligibility criteria, and the sources and methods of selection of participants. Describe methods of follow-up  *Case-control study*—Give the eligibility criteria, and the sources and methods of case ascertainment and control selection. Give the rationale for the choice of cases and controls  *Cross-sectional study*—Give the eligibility criteria, and the sources and methods of selection of participants | 8-9 | Individuals aged 55+ at death, ICD-10-CM codes used : Falls (W00–W19) and Head injuries (S00–S09) |
|  |  | (*b*) *Cohort study*—For matched studies, give matching criteria and number of exposed and unexposed  *Case-control study*—For matched studies, give matching criteria and the number of controls per case | 8-9 | Not applicable (no matched design). |
| Variables | 7 | Clearly define all outcomes, exposures, predictors, potential confounders, and effect modifiers. Give diagnostic criteria, if applicable | 8-9 | HTN/dementia mortality; Predictors: Age, sex, race, region, rural-urban status; Diagnostic codes specified |
| Data sources/ measurement | 8* | For each variable of interest, give sources of data and details of methods of assessment (measurement). Describe comparability of assessment methods if there is more than one group | 8-9 | "CDC WONDER database," "death certificates," urban-rural via "2013 US census" |
| Bias | 9 | Describe any efforts to address potential sources of bias | 8 | "Underestimation due to death certificate reporting," "misclassification," "unaccounted societal shifts" |
| Study size | 10 | Explain how the study size was arrived at | 8-9 | Entire US population (1999-2020) meeting criteria; no sampling |

Continued on next page

| Quantitative variables | 11 | Explain how quantitative variables were handled in the analyses. If applicable, describe which groupings were chosen and why | 8-12 | Age groups (55–64, 65–74, 75–84, 85+), AAMR/CMR calculations, Joinpoint regression for trends |
| --- | --- | --- | --- | --- |
| Statistical methods | 12 | (*a*) Describe all statistical methods, including those used to control for confounding | 8-9 | Joinpoint regression (AAPC/APC), AAMR with 95% CI |
|  |  | (*b*) Describe any methods used to examine subgroups and interactions | 8-9 | Stratified by age, sex, race, region, rural-urban |
|  |  | (*c*) Explain how missing data were addressed | 8-9 | Not addressed. |
|  |  | (*d*) *Cohort study*—If applicable, explain how loss to follow-up was addressed  *Case-control study*—If applicable, explain how matching of cases and controls was addressed  *Cross-sectional study*—If applicable, describe analytical methods taking account of sampling strategy | 8-9 | Not applicable (complete data). |
|  |  | (*e*) Describe any sensitivity analyses | 8-9 | None performed. |
| Results | | | | |
| Participants | 13* | (a) Report numbers of individuals at each stage of study—eg numbers potentially eligible, examined for eligibility, confirmed eligible, included in the study, completing follow-up, and analysed | 9-12 | \|  \| \| --- \|  \| 252,750 \| \| --- \|   deaths (1999-2020); annual counts (e.g.,5631 in 1999 and then 17910 in 2020) |
|  |  | (b) Give reasons for non-participation at each stage | 9-12 | Not applicable (census data) |
|  |  | (c) Consider use of a flow diagram | 9-12 | Not used. |
| Descriptive data | 14* | (a) Give characteristics of study participants (eg demographic, clinical, social) and information on exposures and potential confounders | 9-12 | Age, sex, race, region, rural-urban distributions. |
|  |  | (b) Indicate number of participants with missing data for each variable of interest | 9-12 | Not reported |
|  |  | (c) *Cohort study*—Summarise follow-up time (eg, average and total amount) | 9-12 | Not applicable |
| Outcome data | 15* | *Cohort study*—Report numbers of outcome events or summary measures over time | 9-12 | AAMR/CMR overall and by subgroups (e.g., "AAMR: 9.71 in 1999 and then 19.85 in 2020"). |
|  |  | *Case-control study—*Report numbers in each exposure category, or summary measures of exposure |  |  |
|  |  | *Cross-sectional study—*Report numbers of outcome events or summary measures |  |  |
| Main results | 16 | (*a*) Give unadjusted estimates and, if applicable, confounder-adjusted estimates and their precision (eg, 95% confidence interval). Make clear which confounders were adjusted for and why they were included | 9-12 | Unadjusted AAMR/CMR with 95% CI; no confounder-adjusted estimates. |
|  |  | (*b*) Report category boundaries when continuous variables were categorized | 9-12 | Age groups defined. Rural-Urban classification defined |
|  |  | (*c*) If relevant, consider translating estimates of relative risk into absolute risk for a meaningful time period | 9-12 | Not translated/ No need |

Continued on next page

| Other analyses | 17 | Report other analyses done—eg analyses of subgroups and interactions, and sensitivity analyses | 9-12 | Subgroup analyses (age, sex, race, region, rural-urban); no sensitivity analyses. |
| --- | --- | --- | --- | --- |
| Discussion | | | | |
| Key results | 18 | Summarise key results with reference to study objectives | 13-18 | Discussion summarizes trends, disparities (age, sex, race, geography) linked to objectives. |
| Limitations | 19 | Discuss limitations of the study, taking into account sources of potential bias or imprecision. Discuss both direction and magnitude of any potential bias | 18 | Death certificate inaccuracies, coding errors, suppressed data for small groups, unmeasured confounders. |
| Interpretation | 20 | Give a cautious overall interpretation of results considering objectives, limitations, multiplicity of analyses, results from similar studies, and other relevant evidence | 13-18 | Cautious conclusion: Rising mortality, disparities, need for targeted interventions; contextualized with prior literature. |
| Generalisability | 21 | Discuss the generalisability (external validity) of the study results | 13-18 | US-wide data; disparities highlight external validity gaps (e.g., rural/Southern populations). |
| Other information | |  | | |
| Funding | 22 | Give the source of funding and the role of the funders for the present study and, if applicable, for the original study on which the present article is based | 19 | The author(s) received no financial support. |

*Give information separately for cases and controls in case-control studies and, if applicable, for exposed and unexposed groups in cohort and cross-sectional studies.

**Note:** An Explanation and Elaboration article discusses each checklist item and gives methodological background and published examples of transparent reporting. The STROBE checklist is best used in conjunction with this article (freely available on the Web sites of PLoS Medicine at http://www.plosmedicine.org/, Annals of Internal Medicine at http://www.annals.org/, and Epidemiology at http://www.epidem.com/). Information on the STROBE Initiative is available at www.strobe-statement.org.

**SUPPLEMENTAL TABLE 1. ANNUAL PERCENTAGE CHANGES (APCS) AND AVERAGE ANNUAL PERCENTAGE CHANGE (AAPCS)**

| **Variable** | **Trend Segment** | **Lower Endpoint** | **Upper Endpoint** | **APC (95% CI)** | **AAPC (95% CI)** |
| --- | --- | --- | --- | --- | --- |
| **Overall** | **1** | **1999** | **2007** | **6.5316* (5.6865-7.3834)** | **3.6578* (3.3246-3.9921)** |
|  | **2** | **2007** | **2020** | **1.9280* (1.6524-2.2044)** | |
| **Sex** |  |  |  |  |  |
| **Female** | **1** | **1999** | **2004** | **8.2534* (6.1592-10.3888)** | **3.7689* (3.1272-4.4145)** |
|  | **2** | **2004** | **2010** | **4.0217* (2.4205-5.6479)** | |
|  | **3** | **2010** | **2020** | **1.4486* (0.9828-1.9165)** | |
| **Male** | **1** | **1999** | **2007** | **5.9535* (5.1860-6.7267)** | **3.3961* (3.0929-3.7001)** |
|  | **2** | **2007** | **2020** | **1.8530* (1.6028-2.1039)** | |
| **Race/Ethnicity** | |  |  |  |  |
| **Asian or Pacific Islander** | **1** | **1999** | **2005** | **6.2887* (1.2571-11.5704)** | **2.1785* (0.7843-3.5919)** |
|  | **2** | **2005** | **2020** | **0.5792 (-0.1460-1.3097)** | |
| **Black or African American** | **1** | **1999** | **2020** | **2.4680* (2.0451-2.8928)** | **2.4680* (2.0451-2.8928)** |
| **White** | **1** | **1999** | **2007** | **7.0138* (6.1487-7.8859)** | **3.9862* (3.6433-4.3303)** |
|  | **2** | **2007** | **2020** | **2.1659* (1.8765-2.4561)** | |
| **Hispanic or Latino** | **1** | **1999** | **2020** | **2.0627* (1.6505-2.4766)** | **2.0627* (1.6505-2.4766)** |
| **Census Region** | |  |  |  |  |
| **Northeast** | **1** | **1999** | **2008** | **6.4260* (5.2631-7.6017)** | **3.7137* (3.1723-4.2580)** |
|  | **2** | **2008** | **2020** | **1.7250* (1.1757-2.2772)** | |
| **Midwest** | **1** | **1999** | **2007** | **6.5406* (5.1711-7.9280)** | **3.9651* (3.4172-4.5159)** |
|  | **2** | **2007** | **2020** | **2.4113* (1.9427-2.8820)** | |
| **South** | **1** | **1999** | **2004** | **7.8457* (5.8671-9.8613)** | **3.8493* (3.2433-4.4589)** |
|  | **2** | **2004** | **2010** | **4.4056* (2.8816-5.9521)** | |
|  | **3** | **2010** | **2020** | **1.5809* (1.1586-2.0049)** | |
| **West** | **1** | **1999** | **2005** | **7.2106* (5.6829-8.7605)** | **3.1486* (2.7188-3.5802)** |
|  | **2** | **2005** | **2020** | **1.5672* (1.3058-1.8292)** | |
| **Urban-Rural** | |  |  |  |  |
| **Urban** | **1** | **1999** | **2004** | **7.3982* (5.7201-9.1029)** | **3.5931* (3.0315-4.1577)** |
|  | **2** | **2004** | **2009** | **4.0770* (2.2056-5.9826)** | |
|  | **3** | **2009** | **2020** | **1.6927* (1.3765-2.0098)** | |
| **Rural** | **1** | **1999** | **2007** | **7.6304* (6.6096-8.6610)** | **4.3965* (3.9927-4.8018)** |
|  | **2** | **2007** | **2020** | **2.4548* (2.1153-2.7955)** | |
| **Age Group** |  |  |  |  |  |
| **55-64 years** | **1** | **1999** | **2007** | **3.9346* (2.3547-5.5389)** | **2.6360* (1.9946-3.2814)** |
|  | **2** | **2007** | **2020** | **1.8449* (1.2890-2.4040)** | |
| **65-74 years** | **1** | **1999** | **2005** | **6.4868* (4.7370-8.2658)** | **2.7703* (2.2736-3.2694)** |
|  | **2** | **2005** | **2020** | **1.3203* (1.0104-1.6312)** | |
| **75-84 years** | **1** | **1999** | **2005** | **7.9075* (6.7214-9.1069)** | **3.5090* (2.9935-4.0271)** |
|  | **2** | **2005** | **2010** | **3.6541* (1.8909-5.4478)** | |
|  | **3** | **2010** | **2020** | **0.8857* (0.5081-1.2647)** | |
| **85+ years** | **1** | **1999** | **2008** | **6.8044* (5.7594-7.8598)** | **4.5301* (4.0579-5.0045)** |
|  | **2** | **2008** | **2020** | **2.8563* (2.4163-3.2980)** | |

**SUPPLEMENTAL TABLE 2 SEX STRATIFIED AGE-ADJUSTED MORTALITY RATES PER 100,000 IN ADULTS IN THE UNITED STATES, 1999 TO 2020**

| **Year** | **Men (95% CI)** | **Women (95% CI)** | **Overall (95% CI)** |
| --- | --- | --- | --- |
| **1999** | 13.80 (13.30 – 14.30) | 7.10 (6.83 – 7.38) | 9.71 (9.46 – 9.96) |
| **2000** | 13.70 (13.20 – 14.19) | 6.90 (6.63 – 7.17) | 9.53 (9.28 – 9.78) |
| **2001** | 14.85 (14.34 – 15.36) | 7.55 (7.27 – 7.83) | 10.38 (10.12 – 10.64) |
| **2002** | 16.18 (15.65 – 16.71) | 8.41 (8.12 – 8.70) | 11.42 (11.15 – 11.69) |
| **2003** | 16.91 (16.37 – 17.44) | 9.19 (8.88 – 9.49) | 12.14 (11.86 – 12.41) |
| **2004** | 18.54 (17.99 – 19.09) | 9.99 (9.67 – 10.30) | 13.39 (13.11 – 13.68) |
| **2005** | 19.46 (18.91 – 20.02) | 10.38 (10.06 – 10.70) | 13.93 (13.64 – 14.22) |
| **2006** | 19.62 (19.06 – 20.17) | 10.61 (10.28 – 10.93) | 14.22 (13.93 – 14.51) |
| **2007** | 20.90 (20.34 – 21.46) | 11.44 (11.11 – 11.77) | 15.19 (14.89 – 15.49) |
| **2008** | 21.74 (21.18 – 22.31) | 11.74 (11.40 – 12.07) | 15.74 (15.44 – 16.03) |
| **2009** | 22.27 (21.70 – 22.83) | 11.93 (11.60 – 12.27) | 16.09 (15.79 – 16.39) |
| **2010** | 22.82 (22.26 – 23.39) | 12.70 (12.36 – 13.04) | 16.77 (16.47 – 17.08) |
| **2011** | 22.86 (22.31 – 23.42) | 12.26 (11.93 – 12.59) | 16.60 (16.30 – 16.90) |
| **2012** | 23.07 (22.52 – 23.62) | 12.94 (12.60 – 13.28) | 17.07 (16.77 – 17.36) |
| **2013** | 24.07 (23.52 – 24.62) | 13.16 (12.82 – 13.49) | 17.65 (17.35 – 17.95) |
| **2014** | 24.94 (24.39 – 25.50) | 13.55 (13.21 – 13.89) | 18.30 (18.00 – 18.60) |
| **2015** | 25.00 (24.45 – 25.55) | 13.57 (13.24 – 13.91) | 18.35 (18.05 – 18.65) |
| **2016** | 24.93 (24.39 – 25.47) | 13.91 (13.58 – 14.25) | 18.55 (18.25 – 18.85) |
| **2017** | 25.96 (25.41 – 26.50) | 13.82 (13.49 – 14.15) | 18.92 (18.62 – 19.21) |
| **2018** | 25.97 (25.43 – 26.50) | 14.05 (13.72 – 14.38) | 19.06 (18.77 – 19.35) |
| **2019** | 26.26 (25.73 – 26.79) | 14.21 (13.89 – 14.54) | 19.27 (18.98 – 19.56) |
| **2020** | 27.22 (26.69 – 27.76) | 14.50 (14.17 – 14.83) | 19.85 (19.56 – 20.15) |
| **TOTAL** | 22.1 | 11.77 | 15.93 |
|  | (21.98 –22.22) | (11.7 –11.84) | (15.87 –15.99) |

**SUPPLEMENTAL TABLE 3 AGE-ADJUSTED MORTALITY RATES PER 100,000 STRATIFIED BY RACE IN ADULTS IN THE UNITED STATES, 1999–2020**

| **Year** | **Asian or Pacific Islander (AAMR, 95% CI)** | **Black or African American (AAMR, 95% CI)** | **White (AAMR, 95% CI)** | **Hispanic or Latino (AAMR, 95% CI)** |
| --- | --- | --- | --- | --- |
| **1999** | 11.27 (9.26-13.28) | 6.82 (6.08-7.56) | 9.83 (9.56-10.11) | 8.94 (7.75-10.12) |
| **2000** | 10.90 (8.99-12.81) | 5.91 (5.22-6.6) | 9.75 (9.47-10.02) | 9.96 (8.74-11.18) |
| **2001** | 11.00 (9.15-12.85) | 6.67 (5.94-7.39) | 10.75 (10.46-11.03) | 8.06 (7.01-9.11) |
| **2002** | 14.65 (12.61-16.69) | 6.38 (5.67-7.09) | 11.78 (11.49-12.08) | 9.29 (8.2-10.39) |
| **2003** | 14.20 (12.25-16.15) | 7.01 (6.28-7.75) | 12.52 (12.22-12.83) | 10.68 (9.52-11.84) |
| **2004** | 12.94 (11.14-14.74) | 8.30 (7.51-9.09) | 13.97 (13.65-14.29) | 10.84 (9.69-11.98) |
| **2005** | 16.28 (14.33-18.22) | 7.97 (7.2-8.74) | 14.48 (14.16-14.81) | 12.00 (10.84-13.15) |
| **2006** | 15.18 (13.37-17.00) | 7.94 (7.17-8.7) | 14.91 (14.58-15.23) | 10.94 (9.86-12.03) |
| **2007** | 15.15 (13.41-16.90) | 7.81 (7.06-8.56) | 16.05 (15.72-16.39) | 11.54 (10.45-12.63) |
| **2008** | 15.47 (13.73-17.20) | 7.74 (7.01-8.47) | 16.71 (16.37-17.05) | 12.43 (11.33-13.53) |
| **2009** | 15.87 (14.17-17.58) | 8.38 (7.63-9.14) | 17.10 (16.76-17.44) | 12.47 (11.4-13.55) |
| **2010** | 15.09 (13.47-16.71) | 9.02 (8.24-9.79) | 17.88 (17.53-18.22) | 12.13 (11.11-13.15) |
| **2011** | 17.22 (15.55-18.89) | 8.64 (7.9-9.38) | 17.60 (17.26-17.95) | 12.36 (11.36-13.36) |
| **2012** | 17.42 (15.81-19.03) | 7.93 (7.24-8.63) | 18.18 (17.83-18.52) | 13.85 (12.82-14.88) |
| **2013** | 16.16 (14.67-17.65) | 9.38 (8.64-10.13) | 18.88 (18.54-19.23) | 12.96 (12-13.93) |
| **2014** | 16.55 (15.09-18.00) | 9.25 (8.52-9.98) | 19.67 (19.32-20.02) | 13.40 (12.45-14.35) |
| **2015** | 16.58 (15.18-17.98) | 9.26 (8.54-9.99) | 19.74 (19.39-20.09) | 13.54 (12.61-14.47) |
| **2016** | 16.58 (15.22-17.94) | 10.17 (9.42-10.91) | 19.93 (19.58-20.28) | 13.50 (12.6-14.4) |
| **2017** | 16.08 (14.77-17.38) | 9.28 (8.59-9.98) | 20.60 (20.25-20.95) | 13.51 (12.63-14.39) |
| **2018** | 18.45 (17.1-19.8) | 10.50 (9.77-11.23) | 20.54 (20.2-20.89) | 13.54 (12.68-14.4) |
| **2019** | 16.61 (15.36-17.86) | 10.35 (9.64-11.06) | 20.89 (20.54-21.23) | 14.49 (13.61-15.36) |
| **2020** | 15.89 (14.7-17.08) | 11.32 (10.59-12.05) | 21.62 (21.27-21.97) | 14.79 (13.93-15.65) |
| **TOTAL** | 15.86 | 8.67 | 16.87 | 12.65 |
|  | (15.52 – 16.19) | (8.51 – 8.83) | (16.8 – 16.94) | (12.44 – 12.87) |

**SUPPLEMENTAL TABLE 4 CRUDE RATES PER 100,000 STRATIFIED BY AGE IN OLDER ADULTS, UNITED STATES, 1999–2020**

| **Year** | **55-64 years** | **65-74 years** | **75-84 years** | **85+ years** |
| --- | --- | --- | --- | --- |
| 1999 | 2.58 (2.38–2.79) | 5.75 (5.41–6.10) | 17.08 (16.35–17.81) | 44.99 (42.95–47.03) |
| 2000 | 2.64 (2.43–2.84) | 5.47 (5.13–5.81) | 17.19 (16.46–17.92) | 43.54 (41.56–45.53) |
| 2001 | 2.69 (2.49–2.89) | 5.99 (5.64–6.34) | 19.07 (18.30–19.83) | 46.96 (44.91–49.00) |
| 2002 | 2.69 (2.49–2.88) | 6.70 (6.33–7.07) | 20.77 (19.98–21.56) | 53.54 (51.37–55.71) |
| 2003 | 2.92 (2.72–3.12) | 6.82 (6.44–7.20) | 22.10 (21.29–22.91) | 58.04 (55.80–60.27) |
| 2004 | 3.16 (2.96–3.37) | 7.77 (7.37–8.17) | 24.20 (23.35–25.04) | 63.33 (61.02–65.65) |
| 2005 | 3.22 (3.02–3.42) | 7.81 (7.41–8.21) | 26.04 (25.17–26.92) | 65.54 (63.22–67.86) |
| 2006 | 3.27 (3.07–3.46) | 7.80 (7.40–8.19) | 26.99 (26.10–27.88) | 65.99 (63.71–68.27) |
| 2007 | 3.43 (3.23–3.63) | 8.40 (8.00–8.81) | 27.95 (27.04–28.86) | 73.38 (71.01–75.74) |
| 2008 | 3.39 (3.20–3.59) | 8.31 (7.92–8.71) | 29.52 (28.59–30.45) | 77.00 (74.62–79.39) |
| 2009 | 3.55 (3.35–3.74) | 8.55 (8.15–8.94) | 29.76 (28.83–30.70) | 79.63 (77.24–82.02) |
| 2010 | 3.74 (3.55–3.94) | 8.63 (8.24–9.02) | 30.98 (30.02–31.93) | 84.03 (81.60–86.45) |
| 2011 | 3.72 (3.53–3.91) | 8.46 (8.08–8.84) | 30.50 (29.56–31.45) | 83.46 (81.09–85.82) |
| 2012 | 3.69 (3.50–3.88) | 8.61 (8.24–8.99) | 31.84 (30.88–32.80) | 85.66 (83.29–88.02) |
| 2013 | 3.84 (3.64–4.03) | 8.80 (8.44–9.17) | 32.37 (31.41–33.33) | 90.57 (88.17–92.97) |
| 2014 | 4.16 (3.96–4.36) | 8.81 (8.45–9.17) | 32.67 (31.71–33.63) | 96.36 (93.91–98.81) |
| 2015 | 3.85 (3.66–4.04) | 8.66 (8.31–9.00) | 33.36 (32.40–34.32) | 97.21 (94.78–99.65) |
| 2016 | 4.07 (3.87–4.26) | 9.09 (8.74–9.44) | 32.71 (31.77–33.65) | 99.21 (96.77–101.66) |
| 2017 | 4.07 (3.88–4.26) | 9.30 (8.95–9.65) | 32.87 (31.94–33.80) | 102.79 (100.32–105.26) |
| 2018 | 4.03 (3.84–4.22) | 9.49 (9.15–9.84) | 33.06 (32.15–33.97) | 103.92 (101.45–106.39) |
| 2019 | 4.03 (3.84–4.22) | 9.54 (9.19–9.88) | 33.36 (32.46–34.25) | 105.89 (103.41–108.37) |
| 2020 | 4.62 (4.41–4.82) | 9.81 (9.47–10.15) | 34.24 (33.35–35.13) | 107.02 (104.54–109.51) |
| **TOTAL** | 3.61  (3.57 – 3.66) | 8.32  (8.24 – 8.4) | 28.44  (28.25 – 28.63) | 81.73  (81.22 – 82.24) |

**SUPPLEMENTAL TABLE 5 AGE-ADJUSTED MORTALITY RATES PER 100,000 STRATIFIED BY CENSUS REGION IN ADULTS IN THE UNITED STATES, 1999–2020**

| **Year** | **Northeast** | **Midwest** | **South** |  | **West** |
| --- | --- | --- | --- | --- | --- |
| **1999** | 9.37 (8.83 - 9.91) | 10.50 (9.97 - 11.04) | 8.92 (8.50 - 9.33) |  | 10.38 (9.78 - 10.97) |
| **2000** | 8.70 (8.18 - 9.22) | 10.02 (9.50 - 10.54) | 8.72 (8.32 - 9.12) |  | 11.38 (10.77 - 12.00) |
| **2001** | 9.36 (8.83 - 9.90) | 11.02 (10.48 - 11.57) | 10.09 (9.66 - 10.52) |  | 11.22 (10.62 - 11.82) |
| **2002** | 10.43 (9.87 - 10.99) | 11.92 (11.36 - 12.49) | 10.66 (10.22 - 11.10) |  | 13.26 (12.62 - 13.91) |
| **2003** | 10.79 (10.23 - 11.36) | 12.54 (11.97 - 13.12) | 11.83 (11.37 - 12.29) |  | 13.78 (13.13 - 14.43) |
| **2004** | 12.25 (11.65 - 12.84) | 14.26 (13.65 - 14.87) | 12.70 (12.22 - 13.17) |  | 14.62 (13.96 - 15.29) |
| **2005** | 12.72 (12.12 - 13.33) | 14.60 (13.99 - 15.22) | 13.22 (12.75 - 13.70) |  | 15.73 (15.05 - 16.41) |
| **2006** | 13.14 (12.53 - 13.75) | 15.62 (14.99 - 16.25) | 13.30 (12.83 - 13.77) |  | 15.35 (14.69 - 16.01) |
| **2007** | 14.56 (13.92 - 15.20) | 16.55 (15.90 - 17.19) | 14.07 (13.59 - 14.55) |  | 16.27 (15.60 - 16.95) |
| **2008** | 14.71 (14.07 - 15.35) | 17.28 (16.63 - 17.93) | 14.81 (14.33 - 15.30) |  | 16.55 (15.88 - 17.22) |
| **2009** | 14.82 (14.18 - 15.46) | 17.09 (16.45 - 17.73) | 15.53 (15.04 - 16.03) |  | 17.33 (16.65 - 18.00) |
| **2010** | 15.69 (15.04 - 16.35) | 18.27 (17.61 - 18.93) | 16.25 (15.75 - 16.75) |  | 17.23 (16.57 - 17.90) |
| **2011** | 15.86 (15.21 - 16.52) | 17.45 (16.81 - 18.09) | 16.13 (15.64 - 16.62) |  | 17.24 (16.59 - 17.90) |
| **2012** | 16.47 (15.81 - 17.13) | 17.60 (16.96 - 18.24) | 16.76 (16.27 - 17.26) |  | 17.62 (16.96 - 18.28) |
| **2013** | 17.06 (16.40 - 17.73) | 19.28 (18.62 - 19.94) | 17.18 (16.69 - 17.67) |  | 17.33 (16.69 - 17.97) |
| **2014** | 17.88 (17.20 - 18.56) | 20.20 (19.53 - 20.87) | 17.32 (16.83 - 17.81) |  | 18.30 (17.66 - 18.95) |
| **2015** | 17.32 (16.66 - 17.98) | 19.64 (18.98 - 20.30) | 17.80 (17.31 - 18.28) |  | 18.71 (18.07 - 19.36) |
| **2016** | 16.87 (16.22 - 17.52) | 20.15 (19.49 - 20.82) | 18.22 (17.73 - 18.70) |  | 19.03 (18.38 - 19.67) |
| **2017** | 17.54 (16.89 - 18.19) | 21.00 (20.33 - 21.66) | 18.27 (17.79 - 18.75) |  | 19.01 (18.37 - 19.64) |
| **2018** | 17.87 (17.22 - 18.52) | 21.19 (20.53 - 21.85) | 18.31 (17.83 - 18.78) |  | 19.27 (18.64 - 19.89) |
| **2019** | 18.54 (17.88 - 19.20) | 21.39 (20.73 - 22.05) | 18.24 (17.77 - 18.71) |  | 19.67 (19.04 - 20.30) |
| **2020** | 18.14 (17.50 - 18.79) | 23.44 (22.75 - 24.12) | 18.94 (18.47 - 19.40) |  | 19.51 (18.89 - 20.12) |
| **Total** | 14.81 (14.68 - 14.94) | 17.19 (17.06 - 17.33) | 15.36 (15.26 - 15.46) |  | 16.77 (16.63 - 16.91) |

**SUPPLEMENTAL TABLE 6 AGE-ADJUSTED MORTALITY RATES PER 100,000 STRATIFIED BY URBAN RURAL CLASSIFICATION IN ADULTS IN THE UNITED STATES, 1999–2020**

|  | **Age-Adjusted Rate (95% CI)** |  |
| --- | --- | --- |
| **Year** | **Metropolitan** | **Nonmetropolitan** |
| **1999** | 9.87 (9.58 - 10.15) |  |
| **2000** | 9.8 (9.52 - 10.08) | 8.39 (7.85 - 8.92) |
| **2001** | 10.5 (10.22 - 10.79) | 9.84 (9.27 - 10.42) |
| **2002** | 11.66 (11.35 - 11.96) | 10.47 (9.88 - 11.06) |
| **2003** | 12.36 (12.05 - 12.67) | 11.27 (10.66 - 11.88) |
| **2004** | 13.61 (13.29 - 13.93) | 12.15 (11.52 - 12.78) |
| **2005** | 14.16 (13.84 - 14.49) | 13.09 (12.44 - 13.74) |
| **2006** | 14.36 (14.04 - 14.68) | 13.65 (12.99 - 14.31) |
| **2007** | 15.23 (14.9 - 15.56) | 15.03 (14.34 - 15.72) |
| **2008** | 15.73 (15.4 - 16.06) | 15.73 (15.03 - 16.43) |
| **2009** | 16.21 (15.88 - 16.54) | 15.77 (15.07 - 16.47) |
| **2010** | 16.83 (16.49 - 17.16) | 16.6 (15.89 - 17.32) |
| **2011** | 16.6 (16.27 - 16.93) | 16.45 (15.75 - 17.15) |
| **2012** | 17.12 (16.79 - 17.45) | 16.86 (16.15 - 17.56) |
| **2013** | 17.66 (17.33 - 18) | 17.65 (16.94 - 18.37) |
| **2014** | 18.35 (18.02 - 18.69) | 17.84 (17.12 - 18.55) |
| **2015** | 18.27 (17.94 - 18.6) | 18.42 (17.7 - 19.15) |
| **2016** | 18.38 (18.06 - 18.71) | 19.26 (18.53 - 19.99) |
| **2017** | 18.69 (18.37 - 19.02) | 19.97 (19.23 - 20.7) |
| **2018** | 18.95 (18.63 - 19.27) | 19.63 (18.91 - 20.35) |
| **2019** | 19.16 (18.84 - 19.48) | 19.98 (19.26 - 20.7) |
| **2020** | 19.82 (19.5 - 20.14) | 20.28 (19.55 - 21) |
| **Total** | 16.01 (15.95 - 16.08) | 15.62 (15.48 - 15.77) |

**SUPPLEMENTAL TABLE 7 DEATHS, STRATIFIED BY SEX AND RACE, IN ADULTS IN THE UNITED STATES, 1999 to 2020**

|  |  |  |  | **Deaths** |  |  |  |  |  |
| --- | --- | --- | --- | --- | --- | --- | --- | --- | --- |
| **Year** | **Overall** | **Women** | **Men** | **NH White** | **NH Black or African American** | **NH Asian or Pacific Islander** | **NH American Indian or Alaskan Native** | **Hispanic or Latino** | **Population** |
|  |  |  |  |  |  |  |  |  |  |
| 1999 | 5631 | 2591 | 3040 | 4890 | 329 | 131 | 22 | 234 | 58575867 |
| 2000 | 5617 | 2551 | 3066 | 4891 | 289 | 133 | 19 | 273 | 59266437 |
| 2001 | 6202 | 2818 | 3384 | 5437 | 327 | 144 | 25 | 243 | 60395586 |
| 2002 | 6939 | 3181 | 3758 | 6060 | 318 | 209 | 32 | 296 | 62225539 |
| 2003 | 7522 | 3516 | 4006 | 6539 | 359 | 214 | 43 | 349 | 63872474 |
| 2004 | 8399 | 3872 | 4527 | 7341 | 435 | 209 | 33 | 370 | 65508623 |
| 2005 | 8942 | 4076 | 4866 | 7753 | 421 | 283 | 33 | 441 | 67291295 |
| 2006 | 9286 | 4247 | 5039 | 8102 | 428 | 282 | 43 | 415 | 69094220 |
| 2007 | 10148 | 4660 | 5488 | 8876 | 436 | 303 | 52 | 460 | 70954145 |
| 2008 | 10725 | 4869 | 5856 | 9376 | 452 | 316 | 45 | 520 | 72934684 |
| 2009 | 11221 | 5058 | 6163 | 9753 | 497 | 344 | 49 | 550 | 75028775 |
| 2010 | 11901 | 5450 | 6451 | 10351 | 547 | 346 | 58 | 575 | 76750713 |
| 2011 | 12125 | 5422 | 6703 | 10445 | 553 | 421 | 65 | 618 | 79456281 |
| 2012 | 12760 | 5797 | 6963 | 10946 | 533 | 462 | 61 | 726 | 81731558 |
| 2013 | 13553 | 6042 | 7511 | 11607 | 645 | 466 | 71 | 723 | 84020505 |
| 2014 | 14402 | 6376 | 8026 | 12313 | 656 | 513 | 71 | 802 | 86320792 |
| 2015 | 14717 | 6487 | 8230 | 12528 | 672 | 551 | 59 | 856 | 88638671 |
| 2016 | 15275 | 6787 | 8488 | 12882 | 766 | 587 | 78 | 917 | 90707339 |
| 2017 | 15953 | 6899 | 9054 | 13556 | 726 | 601 | 86 | 946 | 92854337 |
| 2018 | 16488 | 7157 | 9331 | 13788 | 834 | 735 | 100 | 999 | 94703829 |
| 2019 | 17034 | 7365 | 9669 | 14259 | 852 | 691 | 98 | 1106 | 96506800 |
| 2020 | 17910 | 7623 | 10287 | 14916 | 970 | 697 | 108 | 1193 | 98063042 |
| **Total** | 252750 | 112844 | 139906 | 216609 | 12045 | 8638 | 1251 | 13612 | 1.69E+09 |

**SUPPLEMENTAL TABLE 8 MORTALITY, STRATIFIED BY PLACE OF DEATH IN ADULTS IN THE UNITED STATES, 1999 TO 2020**

| **Place of Death** | **Deaths** | | **% of Total Deaths** |
| --- | --- | --- | --- |
| Medical Facility - Inpatient | 158103 |  | |
|  |  | 62.55% | |
|  |  |  | |
| Medical Facility - Outpatient or ER | 16952 |  | |
|  |  | 6.71% | |
|  |  |  | |
| Medical Facility - Dead on Arrival | 742 |  | |
|  |  | 0.29% | |
|  |  |  | |
| Medical Facility - Status unknown | 140 |  | |
|  |  | 0.06% | |
|  |  |  | |
| Decedent's home | 23281 |  | |
|  |  | 9.21% | |
|  |  |  | |
| Hospice facility | 21867 |  | |
|  |  | 8.65% | |
|  |  |  | |

**SUPPLEMENTAL TABLE 9** **AGE-ADJUSTED MORTALITY RATES PER 100,000 STRATIFIED BY STATES IN ADULTS IN THE UNITED STATES, 1999–2020**

| Wisconsin | 24.03 (23.49–24.58) |
| --- | --- |
| Vermont | 22.98 (21.41–24.55) |
| Washington | 21.83 (21.32–22.34) |
| Colorado | 21.81 (21.17–22.44) |
| South Dakota | 20.51 (19.23–21.79) |
| Minnesota | 20.24 (19.71–20.77) |
| Hawaii | 20.14 (19.15–21.13) |
| Utah | 20.13 (19.22–21.04) |
| Rhode Island | 19.89 (18.79–20.98) |
| Kansas | 19.65 (18.95–20.35) |
| New Mexico | 19.53 (18.66–20.39) |
| Iowa | 19.50 (18.86–20.13) |
| Wyoming | 19.16 (17.50–20.83) |
| Maine | 19.04 (18.09–19.98) |
| Idaho | 18.94 (17.94–19.93) |
| Oklahoma | 18.60 (17.99–19.21) |
| Nebraska | 18.48 (17.64–19.32) |
| Arizona | 18.39 (17.92–18.85) |
| Maryland | 18.32 (17.81–18.82) |
| Oregon | 18.25 (17.67–18.83) |
| Pennsylvania | 18.01 (17.71–18.30) |
| Ohio | 17.82 (17.49–18.15) |
| New Hampshire | 17.65 (16.66–18.63) |
| North Carolina | 17.03 (16.65–17.41) |
| Montana | 16.82 (15.75–17.88) |
| Nevada | 16.72 (15.95–17.49) |
| Mississippi | 16.65 (15.98–17.32) |
| North Dakota | 16.56 (15.30–17.82) |
| Georgia | 16.25 (15.84–16.66) |
| Missouri | 16.12 (15.69–16.56) |
| Tennessee | 15.95 (15.50–16.39) |
| Virginia | 15.91 (15.50–16.32) |
| D.C. | 15.83 (14.35–17.30) |
| Florida | 15.80 (15.58–16.02) |
| Massachusetts | 15.74 (15.33–16.14) |
| Michigan | 15.28 (14.95–15.61) |
| Connecticut | 15.11 (14.58–15.64) |
| West Virginia | 15.08 (14.35–15.80) |
| South Carolina | 14.79 (14.29–15.29) |
| Illinois | 14.70 (14.40–14.99) |
| Texas | 14.57 (14.33–14.81) |
| Kentucky | 14.27 (13.77–14.78) |
| California | 14.05 (13.87–14.23) |
| Alaska | 13.91 (12.18–15.64) |
| Delaware | 13.45 (12.41–14.49) |
| Arkansas | 13.40 (12.83–13.97) |
| Indiana | 12.72 (12.33–13.10) |
| New York | 12.72 (12.51–12.94) |
| Louisiana | 11.62 (11.16–12.08) |
| New Jersey | 10.76 (10.47–11.06) |
| Alabama | 9.50 (9.12–9.89) |

**SUPPLEMENTAL TABLE 10 ANNUAL PERCENTAGE CHANGE (APC) OF AGE-ADJUSTED MORTALITY RATES PER 100,000 IN ADULTS IN THE UNITED STATES, 1999 TO 2020**

| **Variable** | **Year Interval** | **APC (95% CI)** | **AAPC (95% CI)** |
| --- | --- | --- | --- |
| Overall | 1999-2007 | 6.5316* (5.6865-7.3834) | 3.6578* (3.3246-3.9921) |
|  | 2007-2020 | 1.9280* (1.6524-2.2044) | |
| Female | 1999-2004 | 8.2534* (6.1592-10.3888) | 3.7689* (3.1272-4.4145) |
|  | 2004-2010 | 4.0217* (2.4205-5.6479) | |
|  | 2010-2020 | 1.4486* (0.9828-1.9165) | |
| Male | 1999-2007 | 5.9535* (5.186-6.7267) | 3.3961* (3.0929-3.7001) |
|  | 2007-2020 | 1.8530* (1.6028-2.1039) | |
| Asian or Pacific Islander | 1999-2005 | 6.2887* (1.2571-11.5704) | 2.1785* (0.7843-3.5919) |
|  | 2005-2020 | 0.5792 (-0.146-1.3097) | |
| Black or African American | 1999-2020 | 2.4680* (2.0451-2.8928) | 2.4680* (2.0451-2.8928) |
| White | 1999-2007 | 7.0138* (6.1487-7.8859) | 3.9862* (3.6433-4.3303) |
|  | 2007-2020 | 2.1659* (1.8765-2.4561) | |
| Hispanic or Latino | 1999-2020 | 2.0627* (1.6505-2.4766) | 2.0627* (1.6505-2.4766) |
| Northeast | 1999-2008 | 6.4260* (5.2631-7.6017) | 3.7137* (3.1723-4.258) |
|  | 2008-2020 | 1.7250* (1.1757-2.2772) | |
| Midwest | 1999-2007 | 6.5406* (5.1711-7.928) | 3.9651* (3.4172-4.5159) |
|  | 2007-2020 | 2.4113* (1.9427-2.882) | |
| South | 1999-2004 | 7.8457* (5.8671-9.8613) | 3.8493* (3.2433-4.4589) |
|  | 2004-2010 | 4.4056* (2.8816-5.9521) | |
|  | 2010-2020 | 1.5809* (1.1586-2.0049) | |
| West | 1999-2005 | 7.2106* (5.6829-8.7605) | 3.1486* (2.7188-3.5802) |
|  | 2005-2020 | 1.5672* (1.3058-1.8292) | |
| Urban | 1999-2004 | 7.3982* (5.7201-9.1029) | 3.5931* (3.0315-4.1577) |
|  | 2004-2009 | 4.0770* (2.2056-5.9826) | |
|  | 2009-2020 | 1.6927* (1.3765-2.0098) | |
| Rural | 1999-2007 | 7.6304* (6.6096-8.661) | 4.3965* (3.9927-4.8018) |
|  | 2007-2020 | 2.4548* (2.1153-2.7955) | |
| 55-64 years | 1999-2007 | 3.9346* (2.3547-5.5389) | 2.6360* (1.9946-3.2814) |
|  | 2007-2020 | 1.8449* (1.289-2.404) | |
| 65-74 years | 1999-2005 | 6.4868* (4.737-8.2658) | 2.7703* (2.2736-3.2694) |
|  | 2005-2020 | 1.3203* (1.0104-1.6312) | |
| 75-84 years | 1999-2005 | 7.9075* (6.7214-9.1069) | 3.5090* (2.9935-4.0271) |
|  | 2005-2010 | 3.6541* (1.8909-5.4478) | |
|  | 2010-2020 | 0.8857* (0.5081-1.2647) | |
| 85+ years | 1999-2008 | 6.8044* (5.7594-7.8598) | 4.5301* (4.0579-5.0045) |
|  | 2008-2020 | 2.8563* (2.4163-3.298) | |

**SUPPLEMENT FIGURE 1.** **FALL-RELATED HEAD INJURY AGE-ADJUSTED MORTALITY RATES BY PLACE OF DEATH PER 100,000 ADULTS IN THE UNITED STATES, 1999–2020**


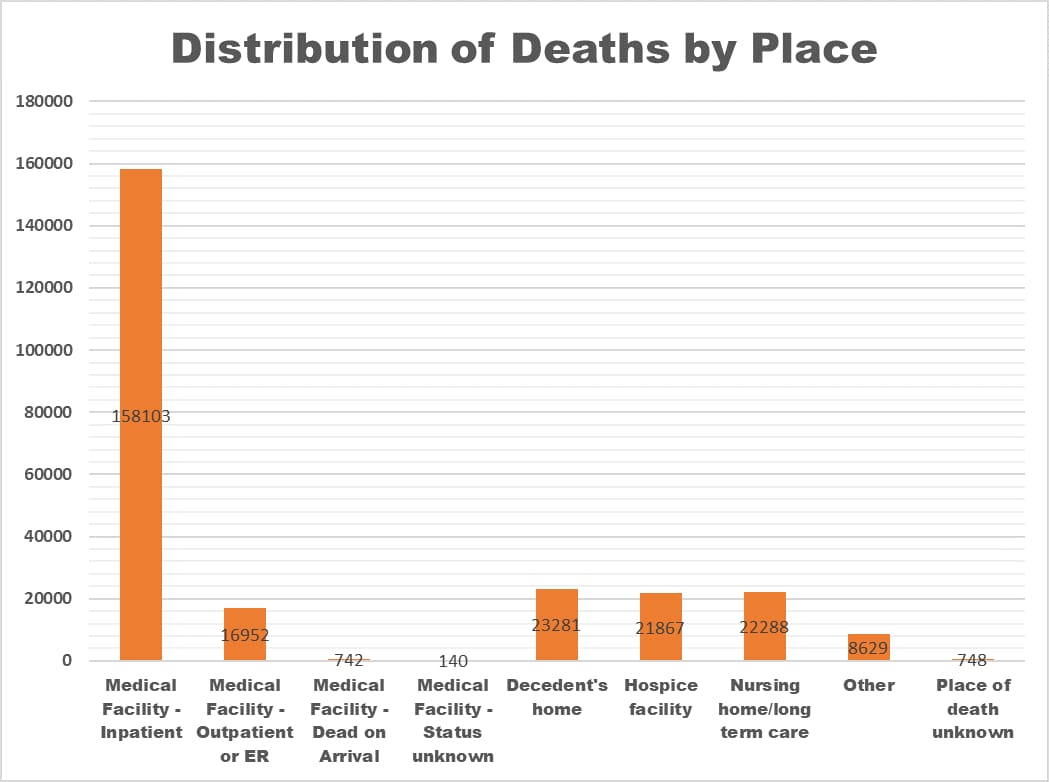


**SUPPLEMENTAL FIGURE 2. STATE-LEVEL FALL-RELATED HEAD INJURY AGE-ADJUSTED MORTALITY RATES PER 100,000 ADULTS IN THE UNITED STATES, 1999–2020 (CHOROPLETH MAP)**


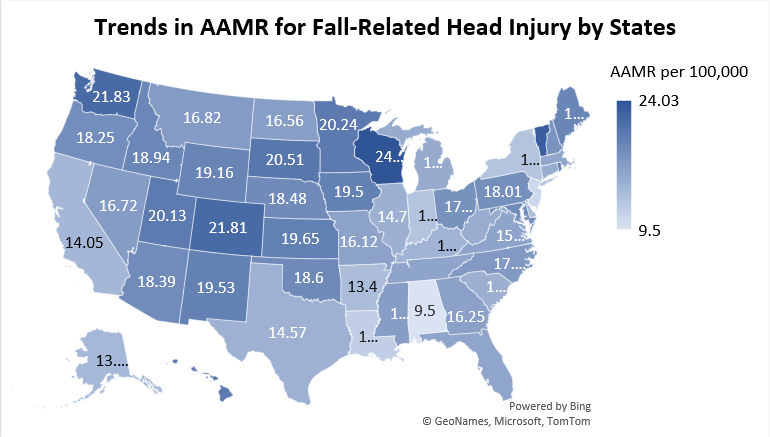

Supplement: Supplementary file 1 — Supplementary Materials: brb371048‐sup‐0001‐SuppMat.docx [file BRB3-15-e71048-s001.docx]
